# Road Performance and Multi-Objective Optimization Study of rWTB—Salt-Retaining Asphalt Mixture

**DOI:** 10.3390/polym17101304

**Published:** 2025-05-10

**Authors:** Zhaoqiang Wang, Zhonglei Zhang, Shaokai Bai, Yanbo Zhao, Yongcheng Ji

**Affiliations:** 1 Qingdao Municipal Engineering Design and Research Institute Co., Ltd., Qingdao 266100, China; wangzq0532@163.com (Z.W.); zhangzhl0532@163.com (Z.Z.); 13176516578@163.com (S.B.); 2 School of Civil Engineering and Transportation, Northeast Forestry University, Harbin 150040, China; yongchengji@126.com

**Keywords:** waste wind power fibers, salt-retaining asphalt mixture, fiber reinforcement, road performance, multi-objective optimization

## Abstract

With the intensification of global climate change, the issue of snow and ice accumulation on roads during winter has become increasingly severe, prompting the widespread application of salt-storage asphalt mixtures in highway construction of alpine regions due to their ability to sustainably release salts for snowmelt. The incorporation of salt-storage fillers significantly compromises the road performance of asphalt mixtures, particularly exacerbating deterioration in low-temperature crack resistance and moisture stability while accelerating pavement distress. Although fiber reinforcement technology has been validated for enhancing asphalt mixture performance, conventional fibers suffer from high production costs and inadequate environmental sustainability. The rapid expansion of the wind energy sector in recent years has generated substantial quantities of retired wind turbine blades (rWTB), posing a global challenge for recycling. This study proposes utilizing rWTB in salt-storage asphalt mixtures and investigates their road performance and underlying mechanisms through experimental analysis. The results demonstrate that rWTB fiber addition markedly improves the mechanical properties of salt-storage asphalt mixtures, yet excessive fiber dosages (>0.3%) induce localized fiber agglomeration, thereby slowing or reversing optimization trends. Given the multi-objective optimization challenge of rWTB fiber incorporation, the Technique for Order Preference by Similarity to Ideal Solution (TOPSIS) algorithm was employed as an optimization tool. In-depth analysis yielded four distinct optimal fiber dosage schemes with performance-oriented priorities: 0.2848%, 0.2903%, 0.2881%, and 0.2882%. These findings provide novel insights for rWTB resource recycling and scientific evidence for enhancing the performance of salt-storage asphalt mixtures.

## 1. Introduction

With the exacerbation of global climate change, the problem of snow and ice accumulation on winter roads has become increasingly severe. Salt-retaining asphalt mixtures [1], which can continuously release salts to melt snow, have been widely applied in highway construction in cold regions. However, to enhance snow-melting efficiency and economic performance, commercial salt-retaining fillers have been extensively studied by researchers and applied in practical engineering, partially or completely replacing traditional mineral powder [2]. This substitution, however, may deteriorate the road performance of the mixture [3]. The release of chlorides, a key snow-melting component in salt-retaining materials, lowers the freezing point of surface water and reduces the adhesion between the pavement and ice/snow, significantly improving the snow-melting and ice-breaking functions. Yet, it also causes certain changes in the service performance of the mixture. For instance, the addition of MFL (Mafilon) material significantly enhances the initial ice-melting performance but negatively impacts all service properties, particularly severely degrading water stability [4]. Similarly, the incorporation of V-260 (Verglimit-260) material as an external additive reduces water stability and low-temperature performance [5], accelerating pavement damage. Research indicates that fiber reinforcement technology can effectively improve asphalt mixture performance [6,7]. For example, polyester fibers enhance rutting and crack resistance through reinforcement effects [8,9]. Polypropylene fibers provide three-dimensional reinforcement for concrete, making it more resilient and durable [10,11]. Adding glass fiber to asphalt mixtures improves material strength, fatigue properties, and ductility. Due to its excellent mechanical properties, glass fiber may offer great potential for asphalt modification [12]. Carbon fibers can improve certain mechanical properties of mixtures, such as flexibility and deformation resistance, and mitigate structural fatigue by enhancing resistance to cracking or permanent deformation [13]. Asbestos fibers can also enhance mixture service performance and are biodegradable. However, due to their unsuitability as long-term reinforcement materials [14], they are no longer used after being identified as hazardous to health [15,16]. Thus, adding fibers to asphalt mixtures has proven to be an effective method to improve performance [17,18].

Nevertheless, traditional fiber production involves high costs and insufficient environmental sustainability [19]. Moreover, existing studies primarily focus on conventional asphalt mixtures, with limited in-depth exploration of salt-retaining fiber-type asphalt mixtures and their synergistic effects.

Recycled wind turbine blades (rWTB) fibers exhibit unique economic advantages compared to traditional fibers. Wind turbine blade (WTB) waste will be estimated to exceed 2 million tons by 2050 [20], making the use of this solid waste material in high-performance salt-retaining asphalt mixtures highly valuable and meaningful for research. Despite the limited studies on the recycling and reuse of rWTB in asphalt pavements in recent years, the similarity in material properties between rWTB and fiber-reinforced polymer (FRP) composites provides a research framework. By drawing on the achievements of FRP-modified asphalt studies, the potential application of rWTB can be better understood. For instance, researchers such as Lin and colleagues [21] used glass fiber-reinforced polymer (GFRP) as a modifying additive in asphalt mixtures. They found that it significantly improved the mixture’s resistance to rutting, fatigue, aging, and water damage, although its performance under low-temperature conditions was less satisfactory. Further studies by Yang and others [22] demonstrated that FRP, as a reinforcing material for asphalt, could optimize high-temperature stability indices (including creep modulus, resistance to permanent deformation, and creep recovery properties) while effectively enhancing the mixture’s ability to resist moisture-induced damage. These studies indicate that, as modifying additives, FRP materials show promising application prospects in asphalt pavements, significantly improving road performance. Based on a comparative analysis of material properties, rWTB should also possess the potential to be utilized as an asphalt modifier for resource recovery.

This study introduces rWTB into salt-storing asphalt mixtures for the first time and systematically investigates the influence patterns of replacing mineral filler entirely on an equal-volume basis with a self-developed low-chloride salt snow-melting filler (Low Chloride Salt Snow-Melting Filler, LCSMF) on the high-temperature stability, low-temperature crack resistance, and moisture susceptibility of the mixture. Furthermore, it innovatively incorporates multi-objective optimization theory to construct a dosage optimization model based on the ideal point method, specifically addressing the multi-dimensional characteristics of pavement performance in salt-storing asphalt mixtures. Traditional studies predominantly employ single-factor analysis methods, which struggle to comprehensively consider the intricate relationships among multiple performance indicators. In contrast, this study utilizes experimental data to establish fitting functions for each performance indicator and applies the ideal point method to determine optimal dosage schemes with different preferences. This systematic research framework not only enhances the scientific rigor of the conclusions but also provides quantifiable dosage design guidelines for engineering applications, demonstrating substantial methodological innovation value.

## 2. Materials and Methods

### 2.1. Materials

The asphalt selected for this study was SBS (Styrene–Butadiene–Styrene)-modified asphalt. Its technical specifications are presented in Table 1. Test results indicated that the asphalt used in this study meets the technical requirements for asphalt in asphalt mixtures as specified in relevant construction standards.

In this study, limestone gravel was used as the asphalt-stabilized macadam aggregates. The coarse aggregate specification was 9.5–13.2 mm, 4.75–9.5 mm, and 2.36–4.75 mm. Fine aggregates consisted of limestone-manufactured sand with particle sizes ranging from 0 to 2.36 mm. The tested properties of the aggregates conformed to the requirements of JTG F40-2004 [23] specifications and the design criteria. Its detailed technical indicators are shown in Table 2 and Table 3 as follows.

In this study, limestone powder was used as the mineral filler, and its technical specifications comply with the requirements of the “Technical Specifications for Construction of Highway Asphalt Pavements” (JTG F40-2004). Table 4 presents detailed parameters.

The self-developed LCSMF (Table 5 below)was used as the salt-storage filler. The porous material was selected as the carrier, and acetate and chloride were used as the main components of snow melting. It has remarkable snow-melting efficiency and good performance. Environmental protection, slow release, and low corrosion in one can partially or completely equal volume to replace the mineral powder in the mixture.

**Figure 1:**
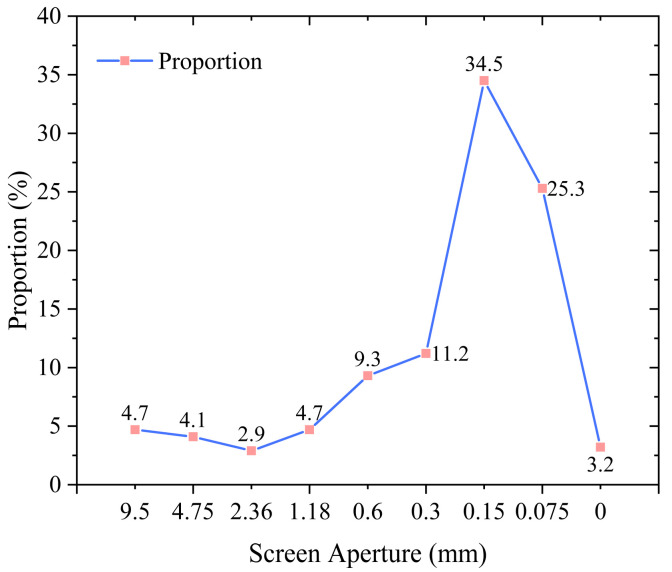
rWTB fiber screening curve.

**Figure 2:**
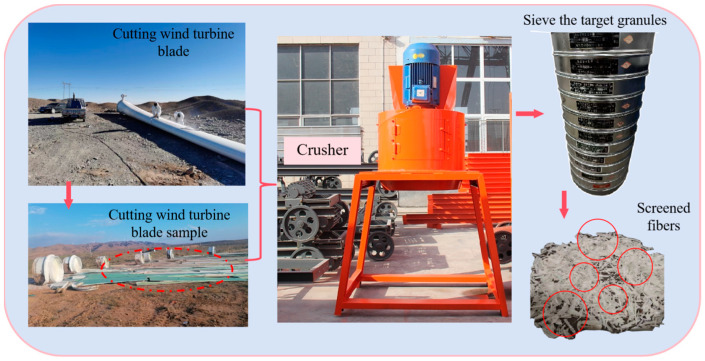
rWTB crushing and recycling process.

The fibers used in this experiment are recycled wind turbine blades broken into fiber form. The surface of the wind turbine fibers is attached with a small amount of epoxy resin, and the fibers are highly pure. Its physical indicators are shown in Table 6, the detailed crushing process is shown in Figure 1, and the screening curve is shown in Figure 2.

### 2.2. Methods

#### 2.2.1. Gradation Design

**Figure 3:**
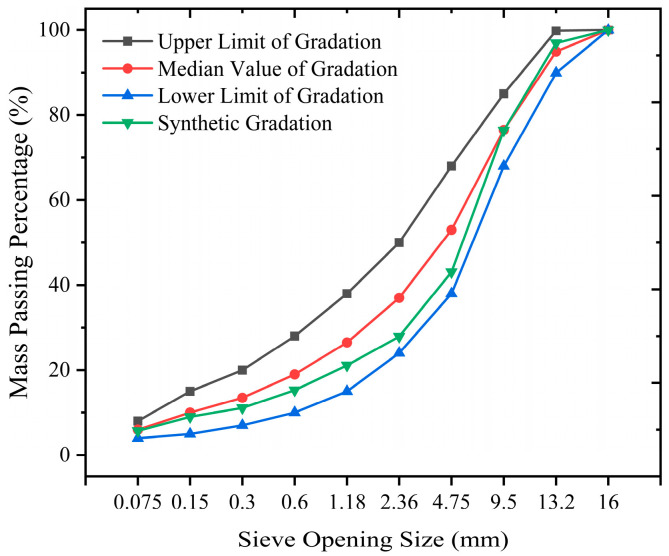
Gradation Curve of AC-13 Asphalt Mixture.

**Figure 4:**
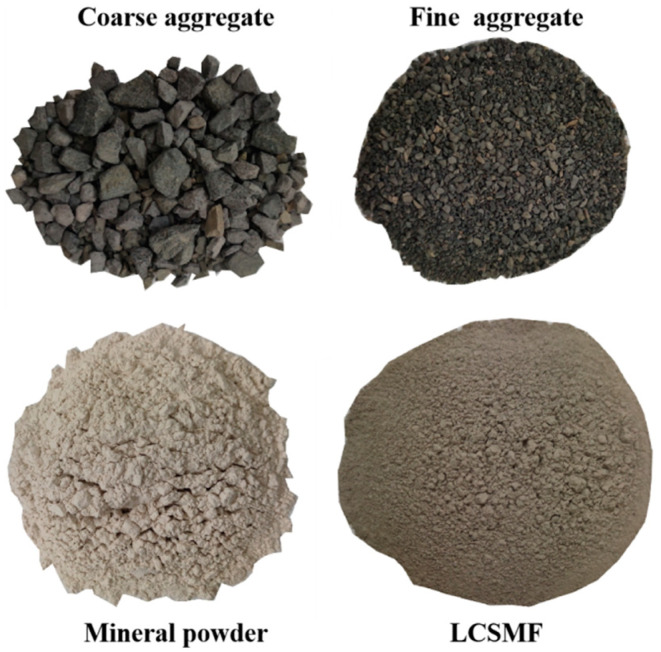
Aggregate Composition Diagram.

The gradation of aggregates used in the asphalt mixture, including the upper limit, median, lower limit, and synthetic gradation, is shown in Table 7. The gradation curves and the aggregate photos of the asphalt mixture are shown in Figure 3 and Figure 4. Based on the Marshall test, the reasonable dosage of the salt-retaining filler is 3–6% of the mixture mass [24]. Considering that an increase in filler content significantly degrades the overall road performance, comparing the effects of fiber-modified asphalt mixtures becomes easier. Therefore, in this experiment, LCSMF was completely substituted (volume-equivalent complete replacement) for the mineral filler in the mixture mass to investigate further the influence of externally added fiber on the road performance of asphalt mixtures.

#### 2.2.2. Preparation and Mixing Process of Fiber Asphalt Mixture

The mixing process of fiber asphalt mixture differs from that of conventional asphalt mixture, with the key of selecting the appropriate timing for fiber addition. Typically, there are two mixing methods available for the preparation of fiber asphalt mixture: the first involves directly mixing the fiber with aggregates and stirring until roughly uniform, followed by adding asphalt and mineral filler for further mixing; the second method entails pre-mixing the fiber with asphalt in a particular proportion to form an asphalt binder, and then proceeding with subsequent operations by the conventional asphalt mixture mixing procedure. However, the latter method is more complex in practical application, requiring precise testing equipment, thereby increasing construction difficulty and cost. Additionally, relevant studies have found that the agglomeration degree of mixtures prepared by the dry method is significantly lower than that of the wet method. Therefore, this study adopts the former method, optimizing the stirring time to ensure uniform dispersion of fibers in the mixture while maintaining their original performance unaffected.

#### 2.2.3. Experimental Methods

When fiber materials modify salt-storage asphalt mixture, LCSMF snowmelt filler replaces all mineral powder in the mixture by equal volume in this study, and rWTB content is designed into four kinds of content: 0.1%, 0.2%, 0.3%, and 0.4%. In addition, a group of ordinary asphalt mixture and a group of salt-storage asphalt mixture (LCSMF completely replaces mineral powder) were designed in this study. Because it is difficult to control the uniform length of rWTB when it is broken, fibers in the length range of 0.075–9.5 mm were selected.

Based on the designed groups, conventional pavement performance tests were conducted, including high-temperature rutting tests, low-temperature beam bending tests, water-soaked Marshall stability tests, and freeze–thaw splitting tests for the salt-retaining fiber-reinforced asphalt mixtures. Furthermore, considering that fiber-reinforced and salt-retaining asphalt mixtures exhibit significant limitations in water stability when used in pavement applications, this study aimed to prioritize investigating the water stability and resistance to dynamic water flushing under summer rainfall conditions. To this end, a high-temperature dynamic water flushing system was specifically designed to evaluate the performance of this asphalt mixture.

1. High-temperature stability

This study utilized the Asphalt and Asphalt Mixture Test Specifications for Highway Engineering (JTG-E20-2011) [25], specifically Test Method T0719, to evaluate the high-temperature stability of asphalt mixtures through a rutting test. The test was conducted at 60 °C with a wheel pressure of 0.7 MPa ± 0.05 MPa. The rut depths, denoted as *d*_1_ and *d*_2_, were measured at 45 min (*t*_1_) and 60 min (*t*_2_), respectively, with precision up to 0.01 mm. If excessive deformation occurred such that the rut depth reached 25 mm before the 60 min mark, the time at which the 25 mm deformation (*d*_2_) was achieved was recorded as *t*_2_, and the deformation at *t*_1_ (15 min prior to *t*_2_) was noted as *d*_1_. The dynamic stability (*DS*) was calculated using the following formula:

(1) $$DS=\frac{(t_{2}-t_{1})\times N}{d_{2}-d_{1}}\times C_{1}\times C_{2}$$

In the formula: 

*DS*—Dynamic stability of asphalt mixture *DS* (times/mm);

*d*_1_—Deformation of the specimen at the test time of 45 min, mm;

*d*_2_—Deformation of the specimen at the test time of 60 min, mm;

*C*_1_—Coefficient of the test instrument type;

*C*_2_—Specimen coefficient, which is 1.0 when the specimen width is 300 mm;

*N*—Test wheel reciprocating rolling speed, usually 42 times/min.

2. Low-temperature crack resistance

This study employs the small beam bending test specified in the “Highway Engineering Asphalt and Asphalt Mixture Test Procedures” (JTG-E20-2011) under Test Method T0715 to assess the low-temperature crack resistance of asphalt mixtures. This test can measure the deformation resistance of asphalt mixtures under specified temperature and load conditions, providing valuable insights into their performance in cold environments.

The test temperature was set to −10 °C ± 0.5 °C, with a 50 mm/min loading rate. The small beam specimens, with dimensions of 250 mm × 30 mm × 35 mm, were prepared by cutting sections from mixtures compacted using a rolling wheel. The following parameters were calculated based on the test results:

(2) $$R_{B}=\frac{3\times L\times P_{B}}{2\times b\times h^{2}}$$

(3) $$\varepsilon_{B}=\frac{6\times h\times d}{L^{2}}$$

(4) $$S_{B}=\frac{R_{B}}{\varepsilon_{B}}$$

In the above formula: 

*R_B_*: Flexural tensile strength of the specimen (MPa);

*ε_B_*: The maximum flexural strain of the bottom beam at failure;

*S_B_*: The bending stiffness at the time of specimen failure (MPa);

*L*—Span of specimen (mm);

*P_B_*—Maximum load of specimen destruction (N);

*b*—Thickness of specimen (mm);

*h*—Test piece height (mm);

*d*—Deflection at the mid-span of the specimen (mm).

3. Water stability

This study employs the water-soaked Marshall stability test (T0709) and freeze–thaw splitting test (T0729) specified in the “Highway Engineering Asphalt and Asphalt Mixture Test Procedures” (JTG-E20-2011) to evaluate the water stability of asphalt mixtures.

a. Water-Soaked Marshall Stability Test:

The water stability of the mixtures is characterized by the residual stability after water soaking. The calculation is performed using the following formula:

(5) $$MS_{0}=\frac{MS_{1}}{MS}\times 100$$

In the formula: 

*MS*_0_—Water immersion residual stability (%);

*MS*_1_—48 h Stability (kN);

*MS*—30 min Stability (kN).

b. The freeze–thaw splitting strength ratio is calculated as an index to evaluate the stability of the mixture water, and the calculation formula is as follows:

R_T1_ = 0.00628P_T1_/h_1_ (6)

R_T2_ = 0.00628P_T2_/h_2_ (7)

T_SR_ = (R_T2_/R_T1_) × 100 (8)

In the formula: 

T_SR_—Freeze–thaw splitting strength ratio (%);

R_T1_—Split tensile strength of specimens without freeze–thaw cycles (MPa);

R_T2_—Split tensile strength of freeze–thaw cycle specimens (MPa);

*P_T_*_1_—Maximum test load of specimens without freeze–thaw cycles (N);

*P_T_*_2_—Maximum test load of freeze–thaw cycle specimens (N);

*h*_1_—Test height of unfrozen and frozen cycle specimens (mm);

*h*_2_—Height of the freeze–thaw cycle specimen (mm).

4. High-Temperature Dynamic Water Flushing Test

**Figure 5:**
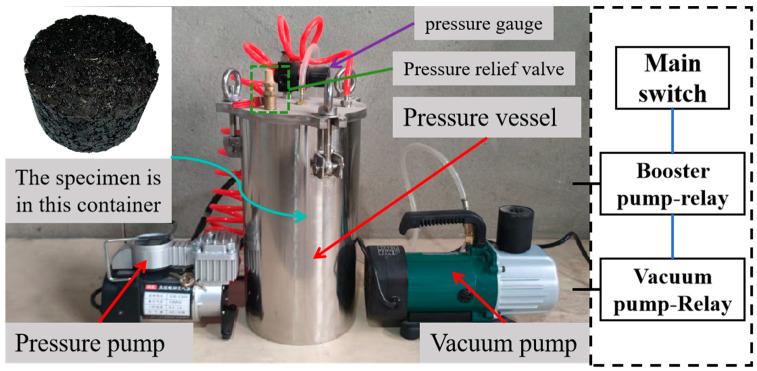
High-Temperature Dynamic Water Scouring Test Apparatus.

The high-temperature dynamic water flushing test is a methodology designed to evaluate the performance of asphalt mixtures under high-temperature and dynamic water conditions. The Figure 5 below shows the self-developed high-temperature dynamic water flushing system. This test simulates the in-service conditions of pavements during summer rainy seasons by subjecting the mixtures to elevated temperatures (50 °C) and cyclic dynamic water flushing. This process mimics the real-world scenario where, after summer rainfall, the pavement surface experiences repeated high-speed traffic, leading to continuous dynamic water pressure and pump suction effects caused by vehicle tires. These conditions subject the asphalt mixtures to cyclic positive and negative pressure changes, accelerating the stripping between asphalt and aggregate and ultimately resulting in pavement distress such as potholes and cracking. Therefore, this test provides a more realistic assessment of material performance under environmental conditions. The operating procedure of this equipment is as follows: First, place the Marshall specimen that has been immersed in water in the pressure chamber, and add water at 50 °C to the chamber to ensure that the water level just covers the specimen. Then, tighten the screws to ensure that the specimen is in the closed reaction device. After that, heat the chamber in a water bath at a constant temperature of 50 °C (to ensure the specimen remains at a constant temperature). Before turning on the power, set the peak positive pressure of the pressure pump and the peak negative pressure parameters of the vacuum pump. Alternating positive and negative pressure once constitutes a complete cycle. The experimental cycle and pressure parameters can be adjusted according to the test requirements.

Salt-storage filler LCSMF is a kind of salt-storage powder filler; its physical properties are similar to mineral powder, so it is used to partially or completely replace mineral powder. However, the road performance of the filler after replacing mineral powder still has obvious defects compared with the actual mineral powder. Therefore, with the increase in the service life of the salt-storage pavement, the porous carrier LCSMF itself and its salt will be lost at a faster rate under the action of repeated erosion and soaking by rain, which will increase the voidage of the mixture, reduce the density and stability of the mixture, and accelerate the damage of the pavement.

Thus, the high-temperature dynamic water flushing test is crucial in this study. Unlike conventional tests that primarily assess the adhesion between aggregates and asphalt, this test evaluates the internal cohesion of the asphalt through dynamic water flushing. This comprehensive approach allows for a more thorough assessment of the water stability of salt-retaining asphalt mixtures. The test enables the investigation of the dynamic water flushing resistance of salt-retaining and fiber-reinforced modified asphalt mixtures, contributing to the optimization of their formulation and design. Ultimately, this optimization enhances the service life and safety of road pavements. The specific procedure of the dynamic water flushing test is as follows:

a. Residual Stability Ratio Test After High-Temperature Dynamic Water Flushing

This method is designed to determine the residual stability ratio of asphalt mixtures before and after high-temperature dynamic water flushing.

Experimental Procedure:

(1) Prepare two Marshall specimens with a diameter of 100 mm for each group, totaling four specimens per group, using double-sided compaction with 50 blows per side. The height of the specimens must comply with 63.5 mm ± 1.3 mm, as specified in JTG E20 Test Method T0702.
(2) Subject the first group of specimens to high-temperature dynamic water flushing at 50 °C and 0.3 MPa for 3500 cycles. Afterward, the stability of the specimens was measured using the JTG E20 Test Method T0709, denoted as MS_1_.
(3) Directly measure the stability of the second group of specimens without dynamic water flushing, denoted as MS.

Data Processing:

Calculate the residual stability ratio of the specimen according to Formula (5), accurate to 0.1%.

b. Variation Rate of Bulk Relative Density After High-Temperature Dynamic Water Flushing

This method is designed to determine the change rate of bulk relative density of asphalt mixtures before and after high-temperature dynamic water flushing.

Experimental Procedure:

(1) Prepare two Marshall specimens with a diameter of 100 mm for each group, totaling four specimens per group, using double-sided compaction with 50 blows per side. The height of the specimens must comply with 63.5 mm ± 1.3 mm, as specified in JTG E20 Test Method T0702.
(2) Measure the bulk relative density of the specimens using JTG E20 Test Method T0705, denoted as ρ_f_.
(3) Subject the specimens to high-temperature dynamic water flushing at 50 °C and 0.3 MPa for 3500 cycles. Afterward, measure the bulk relative density of the specimens again, denoted as ρ_f1_. Calculate the change rate of bulk relative density (Δρ) before and after dynamic water flushing.

Data Processing:

Calculate the change rate of bulk relative density using the following formula, accurate to 0.1%:

Δρ = (ρ_f1_ − ρ_f_)/ρ_f_ × 100 (9)

In the formula: 

Δρ—Change rate of relative density of gross volume after being washed by moving water at high temperature;

ρ_f1_—The relative density of the gross volume of the specimen after being washed by moving water at high temperature (g/m^3^);

ρ_f_—The relative density of the gross volume of the specimen (g/m^3^).

## 3. Results and Discussion

### 3.1. High-Temperature Stability

**Figure 6:**
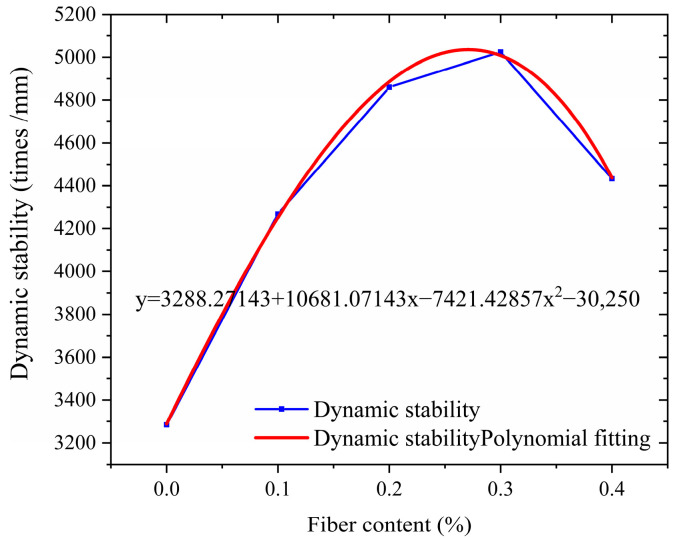
Dynamic stability of asphalt mixtures.

The high-temperature stability of asphalt mixtures can be characterized through rutting tests. This test simulates the condition of asphalt pavements under repeated wheel loads at specific temperatures, and the parameters obtained from the test are derived based on the number of loading cycles and the deformation of the specimens. The rutting test is widely adopted in current research as a practical and straightforward method. This study selects dynamic stability as the technical indicator to evaluate high-temperature performance. The dynamic stability of asphalt mixture specimens at different waste fiber contents is presented in Figure 6.

**Figure 7:**
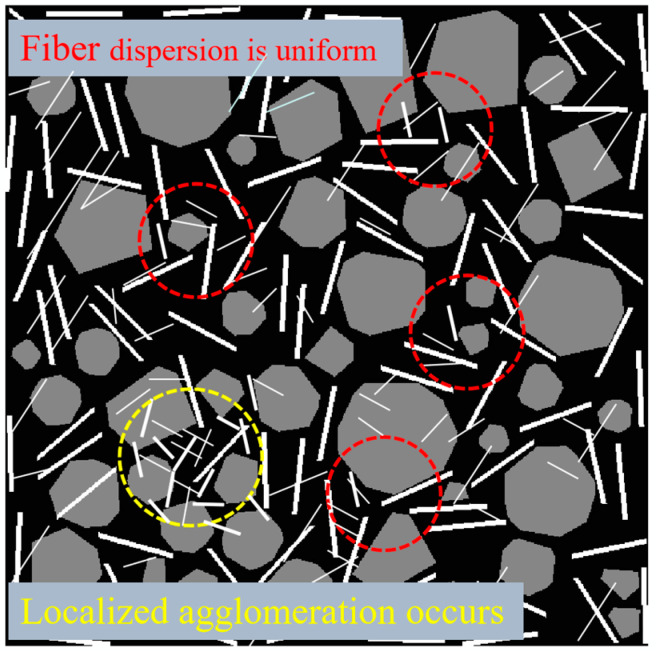
Fiber reinforcement mechanism of the mixture (dosage > 3% rWTB).

As can be seen from Figure 6, with the increase in the content of broken wind fiber, the dynamic stability of the asphalt mixture presents a trend of significant increase at first and then slow decline. The dynamic stability of test groups with 0.1%, 0.2%, 0.3%, and 0.4% was increased by 30.0%, 48.0%, 53.0%, and 34.9%, respectively, compared with the base group (0%). Among them, the 0.3% addition group reached the peak (5025 times/mm), which was 53.0% higher than the benchmark group, and the rutting resistance was the best. When the content exceeds 0.3%, the dynamic stability decreases by 11.8%. It is speculated that an appropriate amount of fiber to ensure uniform fiber dispersion can play a good bridging role, thus enhancing the mechanical properties of the mixture, while excessive fiber leads to uneven dispersion (Figure 7), and fiber aggregation occurs locally, destroying the homogeneity of the asphalt mixture. The research shows that the optimal fiber content needs to balance the reinforcement effect and dispersion uniformity, and the research recommends a 0.2% to 0.3% content range as the reference value for engineering applications.

### 3.2. Water Stability

**Figure 8:**
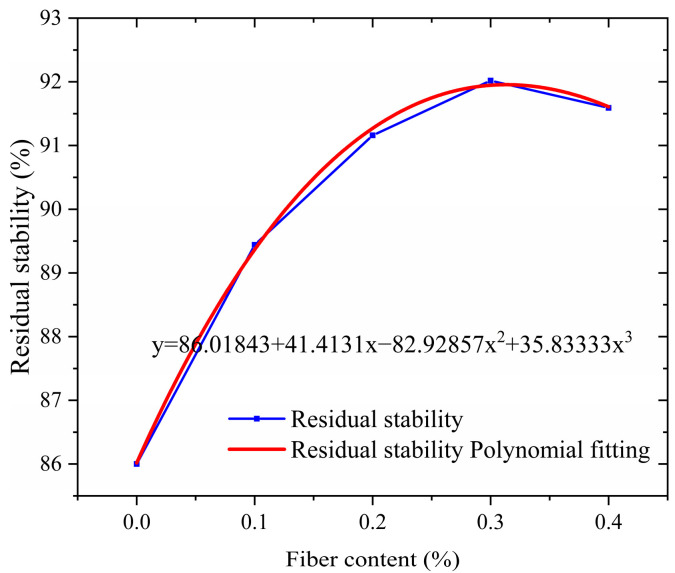
Immersion Residual Stability.

**Figure 9:**
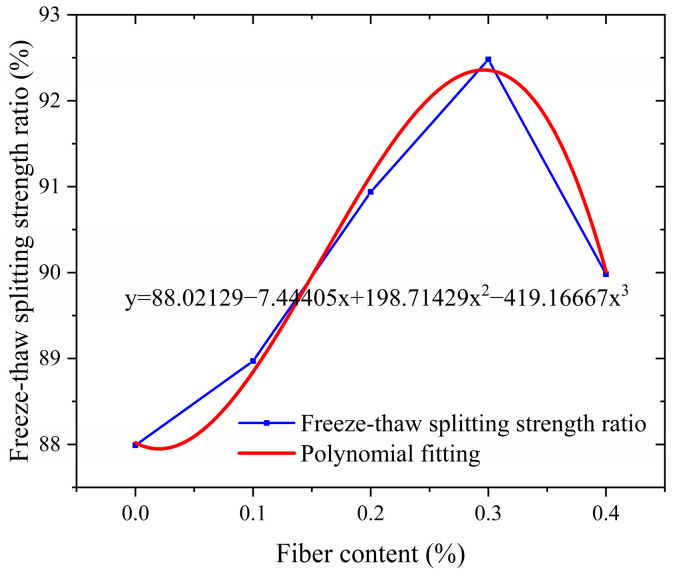
Freeze–thaw fracture strength ratio.

The water stability of asphalt pavement reflects its durability against water erosion. In the actual service process, rainwater infiltration and dynamic water pressure will weaken the bonding strength of the interface between asphalt and aggregate, resulting in water damage such as erosion and loosening of the mixture, which seriously affects the structural integrity of the pavement. In order to scientifically evaluate the water stability performance of the mixture, a dual environmental simulation test scheme was adopted in this study: The immersion Marshall test simulated the infiltration effect of water on the mixture under normal precipitation conditions through the process of vacuum filling water and constant temperature water bath, and focused on evaluating the long-term adhesion stability between the asphalt film and the aggregate. The freeze–thaw splitting test is designed to meet the special requirements of seasonal frozen areas. The freeze–thaw cycle process is set up to reproduce the coupling damage effect of low-temperature frost heave and snow melt seepage, and quantify the water damage resistance of the mixture under extreme climatic conditions. Both test methods were carried out in accordance with standard technical specifications, and the test results are shown in Figure 8 and Figure 9. The water stability characteristics of the mixture in different service environments can be fully revealed by systematically analyzing the variation in performance indexes under different test conditions.

As shown in Figure 8 and Figure 9, the salt-storage asphalt mixture mixed with crushed wind power fibers exhibits a typical three-stage performance evolution law. These three stages are manifested during the process when the fiber content gradually increases from 0% to 0.4%. The road performance shows significant nonlinear response characteristics. The road performance response is slow in the stage of fiber content 0–0.1, and the curve slope is small. The road performance improves rapidly from stage 0.1 to 0.3, and the slope of the curve increases sharply. The road performance tends to stabilize or deteriorate at stage 0.3–0.4, and the curve slope approaches zero or turns negative. Specifically, when the fiber content was in the range of 0% to 0.3%, the residual stability and freeze–thaw splitting strength showed a positive correlation to the two core indexes: the residual stability continued to increase from 86.00% of the base group to 92.02%, an increase of 6.02 percentage points; the ratio of freezing–thawing splitting strength increased from 87.99% to 92.48%, with an increase of 4.49 percentage points (this is because the three-dimensional spatial grid structure formed by adding fibers to the asphalt mixture has a reinforcing and strengthening effect, which can effectively prevent the expansion of crack damage [26]). It is worth noting that at the optimal content point of 0.3%, the two indexes not only reach the peak performance simultaneously, but also form a significant synergistic effect—at this time, the mixture not only maintains more than 92% high-temperature deformation resistance, but also has excellent water damage resistance (T_SR_ > 90%). It fully meets the dual requirements of the JTGD50-2017 [27] specification for a high-grade highway asphalt surface layer.

When the fiber content exceeds the critical value of 0.3%, the performance growth enters the saturation stage and shows signs of attenuation. The residual stability of the 0.4% content group decreased by 0.43% compared with the peak point, and the ratio of freeze–thaw splitting strength decreased by 2.50%. After exceeding the attenuation limit (rWTB > 0.4), excessive fiber dosage will cause fiber agglomeration, and thus its road performance may be further reduced. The specific manifestations can be further studied by expanding the dosage range. This non-monotonic change reveals the complexity of fiber reinforcement mechanism: on the one hand, the appropriate amount of fiber forms a three-dimensional network structure in the asphalt matrix, and dissipates the load energy effectively through “crack deflection” and “bridge action”; on the other hand, excessive fibers lead to local agglomeration, destroy the homogeneity of asphalt mortar, and reduce the interfacial bond strength. From the perspective of the material composite mechanism, the reinforcing effect of wind power fibers stems from their unique physical–chemical coupling effect. The high modulus characteristic of fibers provides rigid support for the asphalt matrix and effectively limits the flow deformation under high-temperature conditions. The fiber diameter is relatively small, with a considerable specific surface area. The fibers are uniformly dispersed in the asphalt, and their huge surface area becomes the wetting interface. At the interface, physical and chemical reactions occur between asphalt and fibers, such as adsorption, diffusion, and chemical bonding. Under this effect, asphalt is arranged in a monomolecular manner on the surface of the fibers, forming a firmly bonded asphalt interface. The asphalt in the interface layer is more viscous and stable than the free asphalt outside the interface layer [28].

### 3.3. Low-Temperature Crack Resistance

Low-temperature crack resistance is a critical performance indicator in evaluating asphalt pavement quality, particularly in northern regions with seasonal permafrost. This property characterizes the ability of pavement materials to resist cracking damage under low-temperature service conditions. The low-temperature small beam bending test, an internationally recognized laboratory testing method, is the preferred approach for assessing the low-temperature performance of asphalt mixtures. This test provides reliable stress–strain analysis, making it a cornerstone for evaluating material behavior under cold conditions. Among the key parameters, the strain at failure serves as a critical quantitative metric, directly reflecting the material’s crack resistance capability under low-temperature environments.

**Figure 10:**
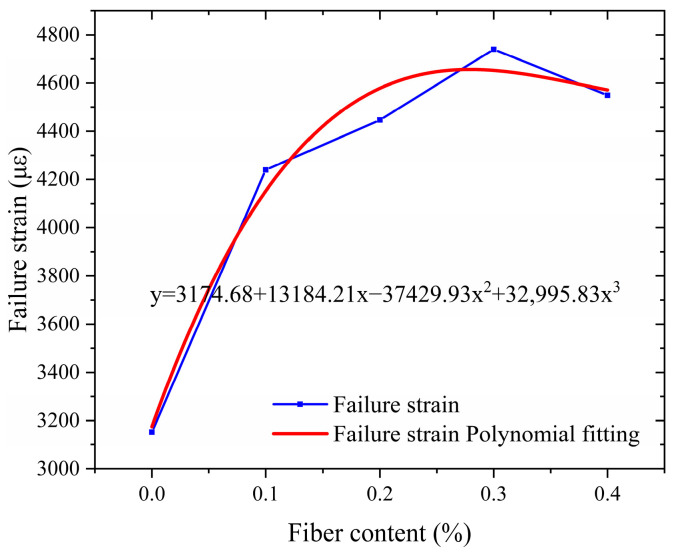
Failure Strain of Asphalt Mixture.

As shown in Figure 10, the failure strain of asphalt mixtures with crushed wind power fiber exhibits an overall trend of increasing and stabilizing with increasing fiber content. Compared to the reference group (0% fiber content), the failure strain for the test groups with 0.1%, 0.2%, 0.3%, and 0.4% fiber content increased by 34.5%, 41.0%, 49.9%, and 44.3%, respectively. Notably, the 0.3% fiber content group achieved the peak value (4739.90 με), with an increase of 49.9%. The addition of fiber significantly improves the low-temperature performance of the mixture through the “crack-bridging” mechanism. On the one hand, the high modulus property of the fiber effectively restricts the low-temperature contraction deformation of the asphalt matrix. (The high modulus characteristic of fibers limits the low-temperature shrinkage deformation of the asphalt matrix because its high strength and high modulus can enhance the overall stiffness of the mixture and resist the shrinkage stress. Fibers form a spatial network structure in asphalt, which disperses stress, reduces the occurrence of cracks. and lowers the amplitude of shrinkage deformation.) On the other hand, the interfacial bonding between the fiber and the SBS-modified asphalt creates resistance to micro-crack propagation. This phenomenon is attributed to fibers’ optimal spatial distribution uniformity at the 0.3% dosage, forming an effective three-dimensional toughening network.

However, when the fiber content exceeds 0.3%, the increase in failure strain decreases by 5.6%, likely due to localized fiber agglomeration caused by excessive fiber content. This leads to stress concentration and reduced toughening efficiency. This study demonstrates that the optimal fiber dosage must balance spatial distribution uniformity and fiber volume fraction. Based on these findings, a dosage range of 0.2% to 0.3% is recommended as a reference for the design of salt-retaining fiber-modified asphalt mixtures.

### 3.4. Performance After High-Temperature Dynamic Water Flushing

Asphalt pavements are subjected to severe challenges to their water stability under the coupled effects of high temperature and dynamic water flow. During summer, high temperatures combined with rainfall and high-speed traffic create repeated positive and negative pressure cycles, leading to the extraction of asphalt mixtures and weakening the interlocking forces and adhesion between aggregates. This results in typical water-induced damage characteristics, such as particle loss and structural instability.

To simulate the conditions experienced by asphalt pavements after rainfall, this study evaluates the residual stability ratio and relative density change rate of asphalt mixtures after 3500 cycles of high-temperature dynamic water flushing under 50 °C and 0.3 MPa conditions. A novel high-temperature dynamic water coupling accelerated testing system was developed and used to assess the degradation rules of water stability for different types of asphalt mixtures. Three compared groups were established: ordinary mixtures, mixtures with snow-melting additives alone, and composite mixtures with both additives and fibers. The goal was to verify whether fiber addition could mitigate the performance losses caused by snow-melting additives, providing technical support for developing novel functional pavement materials with ice removal and durable damage resistance capabilities.

The experimental design groups are as follows: 

Group A: the ordinary mixture;

Group B: the salt-storage filler mixture;

Group C: the salt-storage filler +0.2% fiber mixture;

Group D: the salt-storage filler +0.3% fiber mixture;

Group E: the salt-storage filler +0.4% fiber mixture.

(The above salt-storage filler is used to completely replace mineral filler on an equal-volume basis.)

The experimental study reveals that the salt-retaining asphalt mixture with crushed wind power fiber exhibits significant performance optimization patterns, with its enhancement effects showing nonlinear characteristics as the fiber content varies. The residual stability ratio, a critical indicator of water stability, demonstrates a continuous improvement trend as the fiber content increases from 0% to 0.3%. The 0.2% fiber content group showed an increase of 2.2 percentage points compared to the reference group (fiber-free), rising from 89% to 91%. The 0.3% fiber content group achieved the peak value of 95%, with an increase of 6.7%. However, when the fiber content increased to 0.4%, the residual stability ratio slightly decreased to 94%. This upward and slightly downward trend is also reflected in the relative density change rate, a critical parameter for assessing volume stability. The reference group had a relative density change rate of 0.67%. The 0.2% fiber content group decreased to 0.58% (a reduction of 13.4%). The 0.3% fiber content group reached the lowest value of 0.47% (a reduction of 29.9%). The 0.4% fiber content group slightly increased to 0.53%. A coupled analysis of these two parameters indicates that at 0.3% fiber content, there is a dynamic balance between interfacial bonding reinforcement and volume deformation constraints. The experimental data demonstrate that the crushed wind power fiber can compensate for the localized mechanical losses caused by the salt-retaining additive. Compared to the salt-retaining additive group without fiber (residual stability ratio of 86%, relative density change rate of 0.75%), the 0.3% fiber content group exhibited a 10.5% improvement in water stability (residual stability ratio of 95%) and a 37.3% improvement in volume stability (relative density change rate of 0.47%). These results validate the fiber’s efficacy in repairing physical damage and enhancing material performance, which fully verified the repair efficiency of fiber on physical damage.

**Figure 11:**
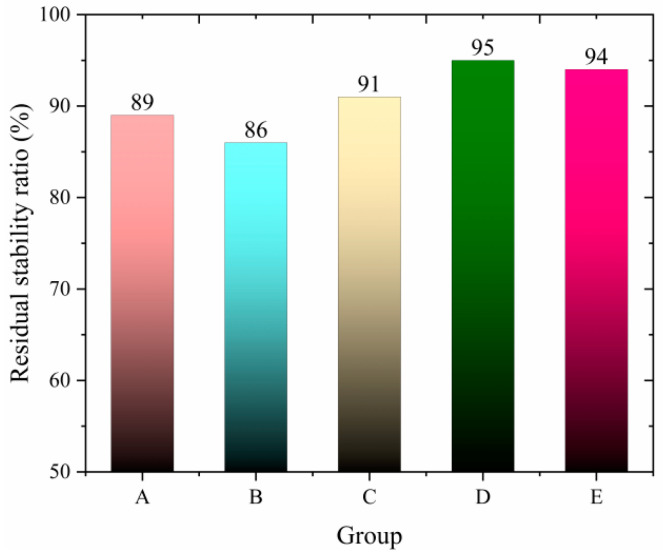
Residual Stability Ratio.

**Figure 12:**
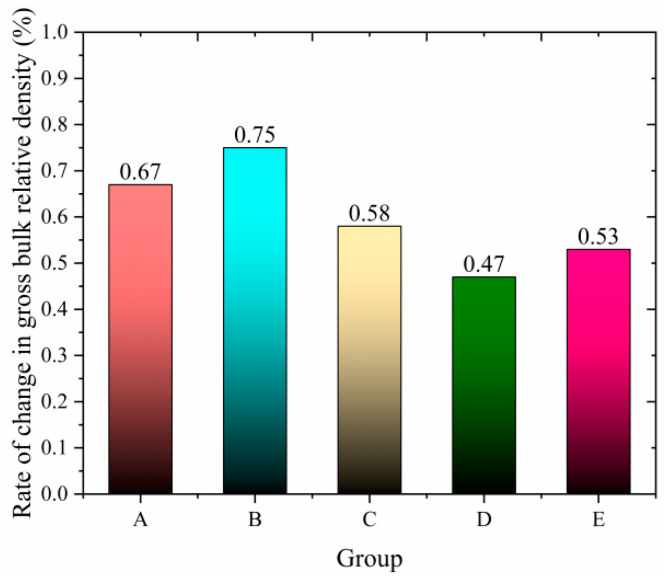
Rate of Change in Bulk Specific Gravity.

Although the two trends in Figure 11 and Figure 12 are opposite, the experiments reflect that the actual situation is that, under the appropriate fiber dosage, the influence of dynamic water scouring is relatively small. The reason is that the wind power crushed fibers may compensate for the local mechanical loss caused by the salt-storage filler. After the fibers are uniformly dispersed in the asphalt, they form a three-dimensional network structure in the asphalt matrix. The bridging effect makes the cementing force between the mixtures stronger, and the mechanical properties are still maintained. This enables the dynamic water scouring test to have a relatively small impact on the residual stability and a lower degree of change in capillary volume.

Therefore, the performance of rWTB asphalt mixture under high-temperature dynamic water erosion is better than that of ordinary asphalt mixture and salt-storage filler mixture, mainly due to the multiple reinforcements of fiber. The fibers form a three-dimensional network structure in the mixture, which limits the movement of mineral particles, enhances the overall stability and crack resistance, and maintains good stability even at high temperatures. At the same time, rWTB can also significantly improve the adhesion between asphalt and minerals, prevent asphalt spalling due to water erosion, improve interface properties, and reduce water seepage. In addition, rWTB optimizes mixture gradation, reduces voids, and increases densification, thereby enhancing impermeability and resistance to water damage. Its high-temperature stability is particularly outstanding; dynamic stability is significantly improved, effectively reducing rutting deformation, keeping the surface of the mixture flat, and further reducing the influence of water erosion. Therefore, rWTB can give full play to its advantages so that the fiber asphalt mixture can perform well under harsh conditions such as high temperature and dynamic water erosion while also solving the problem of construction waste and reducing carbon emissions. This study provides a new way to recycle industrial solid waste and establishes a technical demonstration for the performance restoration of salt-storage asphalt mixture.

### 3.5. rWTB Optimal Fiber Dosage Study

**Figure 13:**
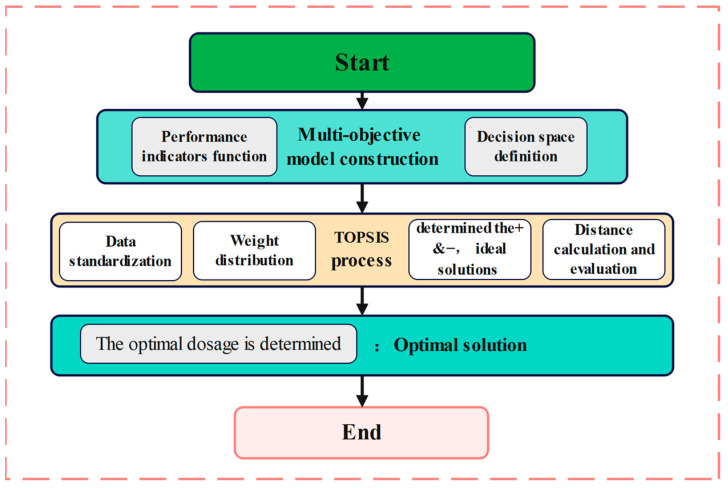
TOPSIS Flowchart.

In multi-objective optimization problems, the Technique for Order Preference by Similarity to Ideal Solution (TOPSIS) is a highly efficient optimization method grounded in multi-objective decision theory (As shown in Figure 13). The core logic of TOPSIS involves quantifying the weighted Euclidean distances between candidate solutions and the ideal solution (a set of global optimal values for all performance indicators) and the negative ideal solution (a set of global worst values for all performance indicators). This process constructs a comprehensive evaluation metric (relative closeness coefficient Ci), enabling the selection of the compromise solution with the best overall performance.

In this study, fiber dosage is treated as the decision variable, while dynamic stability, residual stability, and four other pavement performance indicators are considered optimization objectives. The TOPSIS method is employed to achieve multi-indicator cooperative optimization. This approach quantifies the trade-offs between fiber dosage and its synergistic effects on multiple performance indicators, overcoming the limitations of traditional single-objective optimization. It provides a decision pathway for designing functional asphalt pavement materials that balances mathematical rigor with engineering practicality.

#### 3.5.1. Multi-Objective Optimization Model Construction

TOPSIS (Technique for Order Preference by Similarity to Ideal Solution) method. This approach strictly adheres to the principles of multi-objective decision-making theory and follows a structured process to ensure robust and reliable results.

(1) Functionalization of Performance Indicators

The four critical pavement performance criteria—dynamic stability (*y*_1_), residual stability (*y*_2_), freeze–thaw splitting strength ratio (*y*_3_), and failure strain (*y*_4_)—are expressed as cubic polynomial relationships with respect to the fiber dosage *(x*), where *x* ∈ (0, 0.4). These relationships are derived from experimental data fitting, enabling a quantitative understanding of how fiber dosage influences the performance of asphalt mixtures.

(10) $$\left\{\begin{array}{l} y_{1}=3288.27+10,681.07x-7421.43x^{2}-30,250x^{3}\\ y_{2}=86.02+41.41x-82.93x^{2}+35.83x^{3}\\ y_{3}=88.02-7.44x+198.71x^{2}-419.17x^{3}\\ y_{4}=3174.68+13,184.21x-37,429.93x^{2}+32,995.83x^{3} \end{array}\right.$$

Among them, *x* ∈ (0, 0.4) is the fiber content of broken wind point, *y*_1_ is dynamic stability, *y*_2_ is residual stability, *y*_3_ is freeze–thaw splitting strength ratio, and *y*_4_ is failure strain.

(2) Definition of Decision Space

Since the larger the discreteness, the smaller the step size (*k*) and the more precise the calculation, the fiber content x is discretized into 10,000 candidate schemes; that is, the step size is 1/10,000 (*k* = 1, 2, …, 10000; *x* = 0.00004 k) and a decision matrix *D* is constructed. Each row of D represents a four-dimensional performance vector corresponding to a specific fiber dosage scheme.

#### 3.5.2. Ideal Point Method (TOPSIS) Implementation Process

(1) Data Standardization

The data are standardized using the range method to eliminate dimensional disparities and ensure comparability across performance indicators. Positive indicators (where higher values are preferable) and negative indicators (where lower values are preferable) are treated separately. This step ensures that the optimization process is unbiased and focuses on the relative performance of each candidate solution.

(11) $$z_{ij}\left\{\begin{array}{l} \frac{x_{\mathrm{ij}}-x_{j}^{\min}}{x_{j}^{\max}-x_{j}^{\min}}\left(\mathrm{positive}\;\mathrm{indicators}\right)\\ \frac{x_{j}^{\max}-x_{ij}}{x_{j}^{\max}-x_{j}^{\min}}\left(\mathrm{negative}\;\mathrm{indicators}\right) \end{array}\right.$$

All four indicators in this study are positive indicators.

(2) Weight allocation

The weights were allocated based on the relative importance of the optimization objectives. For the four mix proportioning schemes (high-temperature stability biased, water stability biased, low-temperature cracking resistance biased, and balanced), the weighting vector *W* was respectively assigned as (0.4, 0.2, 0.2, 0.2)^T^, (0.2, 0.3, 0.3, 0.2)^T^, (0.2, 0.2, 0.2, 0.4)^T^, and (0.25, 0.25, 0.25, 0.25)^T^.

Construct a weighted standardization matrix:

(12) V=Z⋅diag(W)

(3) Determination of Ideal and Anti-Ideal Solutions

Ideal Solution: The maximum values of each indicator in the decision matrix.

(13) $$V^{+}=\left[\max(V_{i,1}),\max(V_{i,2}),\max(V_{i,3}),\max(V_{i,4})\right]$$

Negative ideal solution: the minimum value of each index in the decision matrix.

(14) $$V^{-}=\left[\min(V_{i,1}),\min(V_{i,2}),\min(V_{i,3}),\min(V_{i,4})\right]$$

(4) Distance Calculation and Comprehensive Evaluation

For each candidate *i*, the weighted Euclidean distances to the ideal solution and the anti-ideal solution are calculated.

(15) $$D_{i}^{+}=\sqrt{\sum_{j=1}^{4}{\left(V_{ij}-V_{j}^{+}\right)}^{2}},D_{i}^{-}=\sqrt{\sum_{j=1}^{4}{\left(V_{ij}-V_{j}^{-}\right)}^{2}}$$

The relative closeness (*C_i_*) for each candidate scheme *i* is subsequently calculated.

(16) $$C_{i}=\frac{D_{i}^{-}}{D_{i}^{+}+D_{i}^{-}},C_{i}\in \left[0,1\right]$$

A higher value of *C_i_* indicates that solution *i* is closer to the ideal solution and farther from the anti-ideal solution.

#### 3.5.3. Optimal Solution

Optimal decision-making is performed by sorting the *C_i_* values to determine the optimal dosage x*, which satisfies:

(17) $$x^{*}=\underset{x\in (0,0.4)}{\arg\;\max C(x)}$$

**Figure 14:**
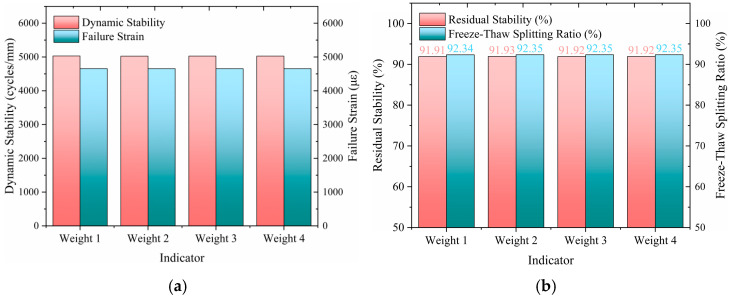
Different weights combine index results: (**a**) High- and low-temperature stability; (**b**) Water temperature stability.

According to the above steps, the optimal dosage scheme with different biases can be obtained, the intuitive results are shown in Figure 14, and the detailed data are presented in Table 8.

Based on the ideal point method for multi-objective optimization analysis, this study designed four weighting schemes targeting the performance requirements of salt-retaining fiber asphalt mixture: high-temperature stability biased ([0.4, 0.2, 0.2, 0.2]), water stability biased ([0.2, 0.3, 0.3, 0.2]), low-temperature cracking resistance biased ([0.2, 0.2, 0.2, 0.4]), and balanced ([0.25, 0.25, 0.25, 0.25]). The results indicate that the high-temperature stability-biased scheme significantly enhanced the dynamic stability index, reaching 5029.5 times/mm (an increase of 0.3% compared to the balanced scheme). However, the comprehensive evaluation score *C_i_* was 0.996, suggesting that overemphasizing a single performance may weaken overall coordination. The water stability bias scheme improved the water stability indices by 0.02–0.03% by increasing the weights of residual stability (91.93%) and freeze–thaw splitting ratio (92.35%). Although the dynamic stability decreased by 1.2%, it exceeded the specification requirements, indicating that the design was reasonable. The low-temperature cracking resistance biased scheme demonstrated the optimal comprehensive performance, achieving a fracture strain of 4655.3 με (close to the maximum value) and a relatively higher *C_i_* of 0.997, reflecting a good balance between crack resistance and multi-objective coordination. The balanced scheme, serving as a benchmark control, maintained high levels in all indices, with an optimal dosage of 0.288% consistent with the low-temperature cracking resistance biased scheme, validating the robustness of the weighting allocation.

The analysis revealed that the optimal dosage fluctuated within the range of 0.285–0.290% (with an extreme difference of only 0.005), indicating a low sensitivity of dosage design to changes in weighting. The influence of different weighting combinations on the results was limited (with a maximum difference in *C_i_* values of 0.001). This may be attributed to the strong correlation among performance indices within the experimental range or a relatively concentrated data distribution, weakening the adjusted effect of weighting. Therefore, this study recommends setting the optimal dosage within 0.285–0.290% for practical engineering applications. If specific performance enhancement is required, expert opinions can be consulted to achieve target-oriented optimization design by fine-tuning weight allocation, following the calculation procedure described above to obtain the optimized design results.

### 3.6. Limitation

Future studies can explore the long-term performance of rWTB pre-storage aggregates in actual use and their impact on road durability. Additionally, the effects of rWTB incorporation on the release rate of salts can be investigated further to promote its widespread application in practical engineering projects.

## 4. Conclusions

In this study, the self-developed salt-storage filler LCSMF was adopted to completely replace the mineral powder in the mixture. The performances of the three types of mixtures, namely, the common type, the salt-storage type, and the rWTB-salt-storage type, were compared emphatically. rWTB was introduced into the salt-stored asphalt mixture for the first time. Its performance was effectively improved by using fiber reinforcement technology. The multi-objective optimization theory was innovatively introduced. Combined with the multi-dimensional characteristics of road performance, a dosage optimization model based on the ideal point method was constructed to obtain the optimal dosage schemes with different biases.

(1) Optimization of rWTB on Road Performance

The rWTB fiber material forms a three-dimensional network structure in the mixture, effectively limiting the loss of mineral particles and improving the high- and low-temperature road performance and water stability of the mixture. The test results show that with the increase in fiber content, the road performance shows a trend of first increasing and then slowly decreasing. However, excessive fiber content (>0.3%) will cause local agglomeration of fibers. This, in turn, affects the performance of the fibers and even deteriorates the toughening effect.

(2) Performance Evaluation After High-Temperature Water Flushing

rWTB can compensate for the local mechanical losses caused by salt-storage fillers. Compared with the salt-storage filler group without fibers (residual stability ratio 86%, density change rate 0.75%), the water stability of the 0.3% group increased by 10.5%, and the relative density of gross volume increased by 37.3%, fully verifying the repair efficiency of fibers on physical damage.

(3) Multi-Objective Optimization Results Under Different Weighting Schemes

Four weighting schemes were analyzed: high-temperature stability-oriented ([0.4, 0.2, 0.2, 0.2]), water stability-oriented ([0.2, 0.3, 0.3, 0.2]), low-temperature anti-cracking-oriented ([0.2, 0.2, 0.2, 0.4]), and balanced ([0.25, 0.25, 0.25, 0.25]). The optimal fiber dosages obtained for these schemes were 0.2848, 0.2903, 0.2881, and 0.2882, respectively. If greater emphasis on a specific weight is desired, expert opinions can be incorporated to adjust the weight parameters, further refining the optimal dosage.

## Figures and Tables

**Table 1:** Technical specifications of the asphalt.

Type of Asphalt	Index	Unit	Test Result	Standard Values
SBS modified asphalt	asphalt penetration (25 °C, 5 s, 100 g)	0.1 mm	76	60–80
	Softening point	°C	63.7	≥55
	Ductility (5 °C, 5 mm/min)	cm	37.4	≥30
	Kinetic viscosity (135°)	Pa·s	1.2	≤3

**Table 2:** Technical specifications and test results of coarse aggregates.

Sieve Size (mm)	Technical Requirement	13.2	9.5	4.75
Apparent relative density	≥2.6	2.783	2.775	2.760
Content of flake (%)	≤15	9.6	10.7	8.3
Water absorption (%)	≤3	0.35	0.72	0.98

**Table 3:** Test results of the technical indexes of fine aggregate.

Sieve (mm)	Technical Specifications	2.36	1.18	0.6	0.3	0.15	0.075
Apparent relative density	≥2.6	2.847	2.724	2.779	2.771	2.720	2.761
Sand equivalent (%)	≥60	96	94	93	97	92	95

**Table 4:** Physical Properties of Limestone Powder.

Test Item		Standard Requirements	Test Result	Test Method
Apparent density (g/cm^3^)		≥2.5	2.693	T0352–2000
Particle size range (%)	0.6 mm	100	100	T0351–2000
	0.15 mm	90–100	93.5	
	0.075 mm	75–100	79.3	
Plasticity index		≤4	2.6	T0354–2000
Hydrophilicity coefficient		<1 (appropriate < 0.8)	0.7	T0353–2000

**Table 5:** Physical Properties of LCSMF.

Test Item	Test Result
Surface	Off-white
Solubility in water	Short-term Insoluble
Density (g/cm^3^)	2.301
Particle Size Range (%)	<0.075 mm
Surface	No agglomerates

**Table 6:** rWTB physical index.

Fiber Type	Elongation at Break/%	Density (g/cm^3^)	Tensile Strength/MPa	Melting Point/°C	Fiber Diameter/µm
rWTB	--	1.57	3770	--	10–15

**Table 7:** Asphalt mixture gradation table.

Sieze/mm	16	13.2	9.5	4.75	2.36	1.18	0.6	0.3	0.15	0.075
Upper limit of gradation	100	99.8	85	68	50	38	28	20	15	8
Median of gradation	100	94.9	76.5	53	37	26.5	19	13.5	10	6
Grade lower limit	100	89.9	68	38	24	15	10	7	5	4
Synthetic gradation	100	96.9	76.4	43.1	27.9	21.1	15.3	11.1	9.0	5.7

**Table 8:** Optimal dosage scheme with different biases.

Index	Weight Comb 1	Weight 2	Weight 3	Weight 4
Optimal dosage (%)	0.2848	0.2903	0.2881	0.2882
Motion stability (times/mm)	5029.5	5023.5	5026.1	5026
Residual stability (%)	91.91	91.93	91.92	91.92
Thaw–freeze crack ratio (%)	92.34	92.35	92.35	92.35
Failure strain; rupture strain (*με*)	4655.8	4654.9	4655.3	4655.3
Relative proximity C_i_	0.996	0.996	0.997	0.996

## Data Availability

The original contributions presented in this study are included in the article. Further inquiries can be directed to the corresponding author.
